# Book Review: Roma Minority Youth Across Cultural Contexts: Taking a Positive Approach to Research, Policy and Practice

**DOI:** 10.3389/fpsyg.2021.736621

**Published:** 2021-08-24

**Authors:** Denisse Manrique-Millones

**Affiliations:** Grupo de Investigación en Comunicación y Salud, Instituto de Investigación Científica, Universidad de Lima, Lima, Peru

**Keywords:** Roma minority, positive youth development, cross-cultural research, public policy, ethnic minority

The book *Roma Minority Youth across Cultural Contexts: Taking a Positive Approach to Research, Policy and Practice* edited by Profs. Radosveta Dimitrova, David Lackland Sam, Laura Ferrer-Wreder at Oxford University Press documents one of the largest marginalized ethnic groups in Europe, the Roma ethnic minority group. The editors focus on a large sample of this underrepresented and consistently stigmatized group from a constructive and positive approach, highlighting the resources, and strengths of children and youth.

Positive Youth Development (PYD) represents one of the most powerful and attractive conceptual frameworks of youth development over the past decades, mainly U.S.-based and applied to middle class, White American and Western European samples (Dimitrova and Wiium, 2021a,b). At the heart of the framework lies the operationalization of PYD as an approach to understand pathways to successful social and individual well-being as to improve a range of developmental competences among young people (Manrique-Millones et al., 2021). In response to this burgeoning knowledge and empirical interest, this book represents a ground-breaking effort to expand the PYD field among ethnic minority youth globally with innovative conceptual and methodological approaches as to provide researchers, policymakers, and practitioners with insights and evidence to optimize health and well-being.

The main models of PYD represented in the volume are the developmental assets (Scales et al., 2017), the 5Cs and 6Cs models of PYD (Geldhof et al., 2015; Burkhard et al., 2019). The developmental assets model emphasizes the alignment between individual strengths, external opportunities, and supports that help youth to achieve optimal development (Scales et al., 2017). The model proposes 40 developmental assets defined as individual strengths and environmental resources, which concern developmental process, experiences, social relationships, contexts, and interactions beneficial for PYD. The 5Cs model developed by several researchers building on one another's work emphasizes internal characteristics of youth that help them to grow into healthy adults namely competence, character, connection and confidence, caring and the 6C of contribution (Geldhof et al., 2015).

Based on these relevant conceptual premises and theoretical foundations, the volume applies PYD by advancing perspectives and impact globally in largely neglected contexts. The chapter authors include leading scholars in PYD and Roma minority studies, developmental and cross-cultural psychology, prevention, intervention, social work, policy, and practice. These authors provide data and evidence from different corners of the world based on their scientific work in their respective countries.

The Roma have idiosyncratic cultural traits and share a common identity, which does not detract from their citizenship, but rather on the contrary, it represents a wealth and added value for the society of which we are all part of. Yet, despite achievements in improving the living conditions of the Roma population, there are still situations that require attention of public efforts and of society as a whole. For example, currently, employment is one of the key aspects for equal opportunity and a fully developed citizenship. Historical reasons, traditions, and ways of life, as well as low levels of education and qualification, have had an influence on the lower-than-average access to employment for the Roma. Likewise, the negative image of the Roma community that persists in the majority of society, with beliefs and prejudices that lead to discriminatory attitudes, continues to be one of the main obstacles preventing them from fully flourish. Each chapter of this book brings one- of- a-kind information about Roma, from a theoretical perspective, based on the strengths and potentials that should be sown in Roma youth, as well as a complement with an empirical perspective, narrating studies in different countries, where it makes us delve more into the subject, formulating implications, and research, policy, and practice.

The book is divided into 12 chapters dedicated to developing a particular theme associated with the Roma ethnic group. The introduction and the first chapters introduce the Roma context describing the socio-cultural characteristics as well as a concise historical frame that leads us to better understand their reality and recognize that oppressed minority groups like Roma have potential and strength than can and should be nurtured and developed.

Chapter 2 presents a comprehensive literature review of the Roma people and youth in United States within the Positive Youth Development perspective (PYD). The chapter transcends the problems that the Roma repeatedly experience by analyzing the processes that can lead to growth and prosperity within the North American context.

Chapter 3 further elaborates on early childhood services to later education performance and outcomes. The chapter addresses the importance of engaging Roma in Provision of Early Childhood and Care in order to promote positive awareness associated to their ethnic identity. In Chapter 4, the authors give a glance of the positive youth development perspective applied to other minority ethnic groups, emphasizing that previous research evidence and intervention in these groups can be adjusted to Roma youth to promote and cultivate their quality of life. Moreover, in Chapter 5, empirical review and supporting evidence on Roma youth is developed, supplemented by empirical illustration in Chapter 6 on developmental assets and thriving indicators, discussing the important role of policies and programs to encourage assets necessary for thriving in school-age sample of Roma youth across Europe.

Chapters 7–10 explore developmental processes of Roma youth based on empirical research in different settings and cultures. From a personal psychological identification such as ethnic identity, self-esteem, self-description domains, to a social based structure such as school engagement and achievement, social connectedness, family closeness, or meaningful friendships with peers. These chapters explore and describe different developmental processes and dynamics as well as person-context interaction that can contribute to an optimal adjustment, under the gaze of a PYD strength-based approach.

In Chapter 11 a critical juncture on the Roma minority group and a reflection on youth development are presented. Finally, in Chapter 12, the PYD perspective effectiveness is evaluated in order to promote relevant recommendations for Roma youth, and likewise, suggestions for well-being and improvement of this ethnic minority group. The primary focus of the concluding chapter by the editors regards lessons learned from applying PYD principles to Roma youth. Unfortunately, there are still developmental gains to be achieved in the midst of prolonged societal and institutionalized discrimination targeting Roma. Taking these circumstances into account, the chapter suggestions appeal for acculturation and multiculturalism such that Roma may finally be integrated into the societies where they reside in.

A major strength of this book is its focus on Positive Youth Development (PYD) among one of the mostly stigmatized and marginalized Roma ethnic minority youth, as the largest at-risk ethnic minority in Europe that is exposed to public intolerance, social exclusion, and poverty. Traditionally, this group is under researched and extremely difficult to reach due to severe discrimination and segregation experiences. Yet, the authors of the volume have been able to reach out to large samples of Roma youth across various countries and apply a PYD approach through a strength-based conception of adolescence. From a conceptual standpoint, this approach applied to these samples provides an important contribution to the understanding of adolescence as much can be gained by documenting the context of development of a marginalized ethnic minority group, such as Roma youth, as well as through the exploration of approaches that can promote well-being.

Further, the volume contributes enormously to the next generation of PYD studies by broadening our knowledge of the link between PYD conditions and optimal outcomes of Roma youth in various countries and regions (e.g., Europe, USA). Such relevant conceptual strength sees a careful documentation of how contextual and socio-political contexts link to well-being of minority youth, spanning variety of disciplines from developmental psychology to cross-cultural and cultural studies, social work, public health, prevention, and intervention.

Beyond the advancement of the theoretical and empirical knowledge base on PYD among ethnic minority groups in global contexts, noteworthy highlights regard the refinement of methodological issues and measurement in under researched contexts (e.g., mixed-methods and structural equations modeling approaches) as well as the integration of PYD scholarship with relevant research, policy, and practice. From a policy and practice standpoints, this volume provides insights on actions to improve life conditions and chances of Roma youth, and potential models for the study of other highly disadvantaged ethnic minority groups by advancing science, practice, and policy relating to youth.

A primary consideration on potential refinements of this volume regards a more inclusive coverage of the psychological experiences, mechanisms, and correlates of PYD among Roma youth in other regions and contexts. For instance, there are many countries hosting Roma settlements that remain uncovered here (e.g., Canada, Asian regions, Western Europe). Future work is needed in these contexts despite the challenges of reaching out and researching Roma populations. Another point pertains to additional methodological approaches beyond cross-cultural, multi-national, and mixed-methods reported in the volume. It is worth noting that despite the variability in methodological approaches, the contributions represent cross-sectional and correlational data thereby limiting the causal inferences on the reported findings. Future work may employ longitudinal and experimental designs are to uncover relevant developmental processes on how PYD develops and changes from adolescence to emerging adulthood and young adulthood among Roma. An advancement of PYD scholarship among Roma populations may also be inclusive with participants from diverse cultural, ethnic, and socio-demographic backgrounds, and comparative samples of mainstream non-Roma youth.

I highly recommend the book *Roma Minority Youth across Cultural Contexts: Taking a Positive Approach to Research, Policy and Practice*, to researchers, practitioners, policy makers, students and professionals interested in knowing more about this fascinating ethnic group, their beliefs, lifestyle, customs, offering an up-to date evidence aiming at promoting Roma youth strengths from a PYD perspective.

## Author Contributions

The author confirms being the sole contributor of this work and has approved it for publication.

## Conflict of Interest

The author declares that the research was conducted in the absence of any commercial or financial relationships that could be construed as a potential conflict of interest.

## Publisher's Note

All claims expressed in this article are solely those of the authors and do not necessarily represent those of their affiliated organizations, or those of the publisher, the editors and the reviewers. Any product that may be evaluated in this article, or claim that may be made by its manufacturer, is not guaranteed or endorsed by the publisher.

## References

[B1] Burkhard B. M. Robinson K. M. Murray E. D. Lerner R. M. (2019). The positive youth development perspective, in The Encyclopedia of Child and Adolescent Development, eds Hupp S. Jewell J. (Hoboken, NJ: Wiley-Blackwell), 1–12.

[B2] Dimitrova R. Wiium N. (2021a). Handbook of positive youth development: Advancing the next generation of research, policy and practice in global contexts, in Handbook of Positive Youth Development. Advancing Research, Policy and Practice in Global Contexts, eds Dimitrova R. Wiium N. (Cham: Springer). Available online at: https://www.springer.com/gp/book/9783030702618

[B3] Dimitrova R. Wiium N. (2021b). Handbook of Positive Youth Development. Advancing Research, Policy and Practice in global Contexts. Cham: Springer. Available online at: https://www.springer.com/gp/book/9783030702618

[B4] Geldhof G. J. Bowers E. P. Mueller M. K. Napolitano C. M. Callina K. S. Walsh K. J.. (2015). The Five Cs model of positive youth development, in Promoting Positive Youth Development. Lessons From the 4-H Study, eds Bowers E. P. Geldhof G. J. Johnson S. K. Hilliard L. J. Hershberg R. M. Lerner J. V.. (Cham: Springer) 161–186.

[B5] Manrique-Millones D. Wiium N. Pineda-Marín C. Fernández-Arata M. Alfonso-Murcia D. López-Martínez J. L.. (2021). Association between substance use behaviors, developmental assets and mental health: a Glance at Latin American Young College students. Front. Psychol. 12:639578. 10.3389/fpsyg.2021.639578 33716908 PMC7947607

[B6] Scales P. C. Roehlkepartain E. C. Shramko M. (2017). Aligning youth development theory, measurement, and practice across cultures and contexts: lessons from use of the Developmental Assets Profile. Child Indic. Res. 10, 1145–1178. 10.1007/s12187-016-9395-x

